# The concept of HRQoL for patients on hemodialysis in Saudi Arabia: an exploratory study

**DOI:** 10.1186/s12955-021-01906-6

**Published:** 2021-12-24

**Authors:** Rima Saleem AL Garni, Mary Cooke

**Affiliations:** 1 Fundamentals of Nursing Department, College of Nursing, Imam Abdulrahman Bin Faisal University, P.O. Box 1982, Dammam, 31441 Kingdom of Saudi Arabia; 2 Urgent and Emergency Nursing, Division of Nursing, Midwifery and Social Work, The University of Manchester, University Place, Oxford Road, Manchester, M139PL UK

**Keywords:** Health-related quality of life, End-stage renal disease, Renal failure, Hemodialysis, Conceptualization, Measurement

## Abstract

**Background:**

The concept of health-related quality of life (HRQoL), a patient-reported outcome measure, is poorly defined within the Saudi literature. There is a lack of culturally adapted measures to assess the HRQoL of patients on hemodialysis in Saudi Arabia. Hence, this study aims to explore and define the concept of HRQoL, identify its key domains and develop a conceptual model as perceived by patients with renal failure who are undergoing hemodialysis in Saudi Arabia.

**Methods:**

Qualitative research methods was used; data were collected in one dialysis center in the Eastern Region of Saudi Arabia. Twenty-two semi structured qualitative interviews were conducted using a topic guide. The data were analyzed using thematic analysis methods as the transcripts were coded, the categories identified, and the themes generated.

**Results:**

Seven definitions of the HRQoL concept emerged from data analysis in terms of health status and psychological wellbeing including the satisfaction with life, socialization and the ability to play the expected social role and having social relationships that are supportive, religiosity and the belief in God and being able to perform religious worships and finally needs satisfaction was used to define HRQoL which included financial needs and the quality of healthcare services. All these themes were utilized to develop one common definition that emphasized the personal satisfaction with health, social, psychological and financial needs in addition to religious performance and the quality of healthcare services provided. The conceptual model was developed using five key domains of HRQoL: physiological, social, psychological, religious and vocational domains that were defined by certain indicators and the relationships between the domains were clarified in the model.

**Conclusion:**

The findings of this study could guide the selection of the appropriate HRQoL instrument to assess the HRQoL of patients on hemodialysis in Saudi Arabia, which would ensure the validity of the findings that could be used in healthcare decisions and planning of care.

**Supplementary Information:**

The online version contains supplementary material available at 10.1186/s12955-021-01906-6.

## Background

The number of patients with end-stage renal failure in Saudi Arabia is increasing yearly as the Saudi Center for Organ Transplantation had reported that the average net annual increase of patients on hemodialysis is 6% [1]. The disease is life-threatening and requires hemodialysis treatment, which has significant effects on the patient’s life. Many physical, psychological and social aspects of the patient’s life are affected by the disease and its treatment [2]. Healthcare providers are challenged when managing patients on hemodialysis. The deteriorating health status and the associated consequent fluctuations in psychological status and failure to fulfil social commitments may interfere with patients’ wellbeing. The HRQoL of patients on hemodialysis is imbalanced and influenced by the patient’s health condition [3]. This may be the case for patients in Saudi Arabia. However, such conclusions cannot be made until the HRQoL is assessed using valid instruments. To date, there is no existing QoL instrument that measures the HRQoL of patients undergoing hemodialysis designed for patients in Saudi Arabia. Additionally, the concept of QoL is highly subjective and is affected by the social characteristics of the assessed individuals within their cultural context. Hence, each society requires valid instruments for assessing HRQoL. In Saudi Arabia, the HRQoL instrument for patients on hemodialysis has not yet been developed. A generic tool, the SF-36, had been culturally adapted for Saudi citizens [4], but it is not a disease-specific tool, and its use for patients with end-stage renal disease on hemodialysis might not reveal disease-specific findings that could help in individualizing healthcare services based on patients’ needs and priorities.

Furthermore, the meaning of the concept of HRQoL in Saudi Arabia is not yet clear [5]. Such an understanding of the concept, its domains and determining indicators and conceptualization is still under investigation. Hence, the current study is designed to achieve the first step of developing/adapting an HRQoL instrument for patients with ESRD undergoing hemodialysis in Saudi Arabia. The meaning of the HRQoL concept and determining its key domains were identified, and further conceptualization of the concept was conducted.

## Methods

### Design and participants

This exploratory study aims to explore the concept of HRQoL as perceived by patients undergoing hemodialysis in Saudi Arabia. To achieve that aim, qualitative methods were used to collect and analyze the data. There is a lack of clear understanding of the HRQoL concept as perceived by patients undergoing hemodialysis in Saudi Arabia. The qualitative methodology provided necessary information to define and conceptualize the HRQoL concept in Saudi Arabia.

A purposive sample of 22 participants who underwent hemodialysis for a minimum of three months was recruited based on the inclusion and exclusion criteria (Table 1). Maximum variation was achieved by recruiting patients from different age groups, levels of education and socioeconomic statuses. This variation was considered to provide different views and opinions about the meaning of HRQoL. The participants were selected from the patients lists after discussion with the haemodialysis charge nurse after explaining the selection criteria. The unit charge nurse introduced the researcher to the selected patient with explanation of the study aim and objectives.

**Table 1:** Inclusion/exclusion criteria

Inclusion criteria	Exclusion criteria
Diagnosed with ESRD (chronic renal failure stage 5)	Patients with end-stage renal disease who are treated with peritoneal dialysis or renal transplantation
Undergoes hemodialysis for at least 3 months	Patients with terminal illness (e.g., cancer)
Lives within the Saudi culture. In addition to Saudis, people from neighboring countries (e.g., Yemen, Iraq and Arabian Gulf countries) who live in Saudi Arabia but are not ethnically Saudis and do not hold a Saudi nationality were included	Patients with communication barriers (e.g., aphasia, hearing loss and non-Arabic and non-English speaker)
Age between 21 and 65 years old	Patients with cognitive impairment

### Data collection 

Individualized semi structured interviews were conducted face-to-face, guided by an interview topic guide (Table 2). The topic guide (interview schedule) included general questions that were used during the interview to allow wide and deep exploration of the HRQoL concept, as it included probes to explore the participant’s responses as needed. The questions were developed based on the study objectives and guided by literature reviews on the topic. Follow-up questions and probes encouraged participants to provide specific comments related to the impact of their disease on their HRQoL. Participants were asked open-ended questions about the effect of kidney failures and haemodialysis on their life, the most affected aspects of their life by the disease, the meaning of quality of life, and its components and what would give their life quality (Additional files 1 and 2).

**Table 2:**
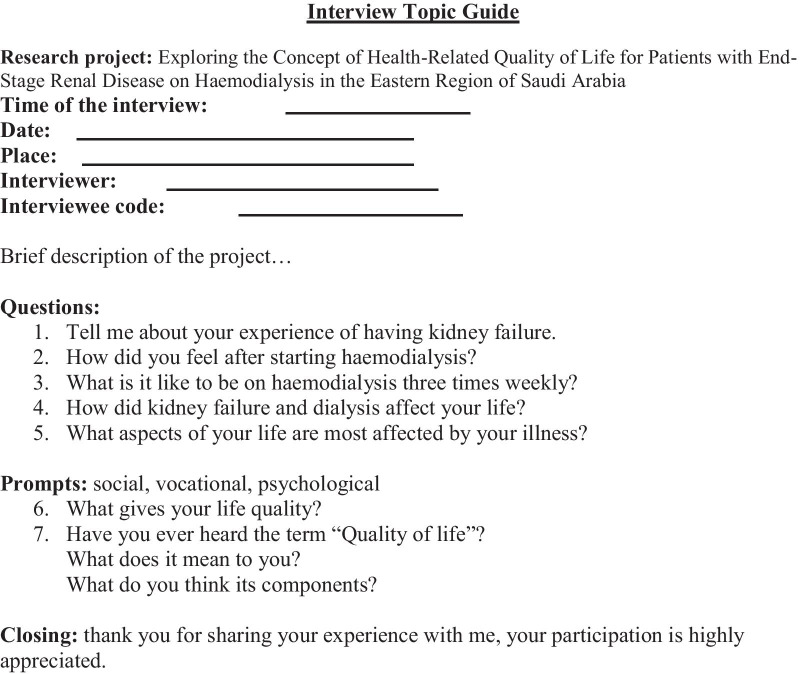
Interview topic guide

The data collection took place during each participant’s hemodialysis procedure in the dialysis unit because the individuals preferred to be interviewed during their hospital visits rather than at home. Privacy was maintained as the curtains were drawn around the dialysis chair and the interview was between the interviewer and the participant only. The local language in Saudi Arabia is Arabic; hence, the interviews were conducted in the Arabic language and were audio-recorded. The interview durations ranged between thirty minutes and two hours. Data analysis was congruent with data collection, which continued until thematic saturation was achieved. Thematic saturation was established during data analysis as the initial codes and themes were generated and when those themes were repeated during the interviews, data saturation was achieved, hence data collection was stopped at this stage.

### Qualitative data analysis

Data analysis included data preparation, which involved verbatim transcription of the recordings, translation into English by a professional translator specialised in health-related translations and backward translation into Arabic to ensure validity of translation and increase the rigor of the study. English translation was needed as the results will be used to develop the conceptual model that will be compared and contrasted with the international QoL models.

Qualitative data analysis was performed using the naturalistic thematic analysis method, following Braun and Clarke [6].

The English transcripts were coded line by line by the first author and revised by the second author. Another independent reviewer, who was the supervisor of the PhD project, had a second revision of the code book. Discussions and meetings took place to resolve discrepancies. The independent reviewer attended the analysis meetings and revised the coding procedure and resulting codes, categories and themes. In case of discrepancy, the literature was approached, listening to the interviews recordings again and re-reading the transcripts to gain understanding and familiarize the researchers with the data. Initially, a total number of 428 codes where identified. Irrelevant codes with the study objectives such as the cause of kidney failure and peritoneal dialysis experience were deleted. The remaining 319 codes were categorized and themes were generated (Table 3). QSR NVIVO 10 was used to store the data and to organize the analysis procedure (Additional file 2).

**Table 3:** Generated themes from data

Study themes	
Physiological domain	
Vocational domain	
Psychological domain	
Healthcare services	
Religious domain	
Illness restriction	
Consequences of illness	
Conceptualisation of QoL	
Financial domain	
Social domain	
Environmental weather	
Control	
Hopes	
Recreation	

### Ethics

This study was governed by ethical approval from the University of Manchester Ethics Committee and the Saudi Ministry of Health General Directorate of Medical Research. Participants were asked to provide written and verbal consent to participate in the study, and their interviews were treated confidentially. The participants’ psychological distress was assessed, and a distress policy was used when needed; lone working policy was applied.

### Rigor of the study

In order to utilize the naturalistic inquiry approach, the researcher undertook training programs on conducting qualitative research methods, semi-structured interviews and the use of QSR NVIVO 10. Additionally, the Quality Framework for Qualitative Research by Meyrick [7] was used to guide the research process and ensure a robust study will be conducted. Peer debriefing during the data collection and analysis ensured neutrality and minimizing bias during data collection and analysis. Furthermore, Backward translation of the transcribed interviews was performed by a professional translator in order to check the validity of the translation. Credibility and confirmability were achieved in the current study by audio-recording the interviews and notes taking. Dependability was ensured through keeping diaries during data collection and analysis. In addition to code-recode procedure [8] was performed as the interview transcripts were coded using paper and pen and repeated when uploading the transcripts into the QSR NVIVO 10 software. Categories and themes were developed by the researchers and revised by an independent researcher, who is expert in quatitative research methods (Professor Ann-Louise Caress) [9]. This is to validate the thematic analysis of the study.

## Results and findings

Twenty-two participants of patients undergoing hemodialysis participated in the study. They varied in age, gender and other physiological and social circumstances, as illustrated in Table 4.

**Table 4:** Sample characteristics

Criteria		N = 22
Age	23–65 years	
Gender	Male	13
	Female	9
Marital status	Single	4
	Married	15
	Divorced	2
	Widowed	1
Working status	Works in a full-time job	9
	Retired	3
	Unemployed	10
Duration of dialysis	7 months–30 years	

### Interpretation of HRQoL

Several definitions emerged from the data analysis (Table 5). These definitions were synthesized after abstracting participants’ responses on the question about the definition of HRQoL. This illustrates the individuality and subjectivity of the concept. These definitions varied according to the participant’s age, level of education and cultural context. Using participants’ words, the authors defined the concept from the seven perspectives identified in Table 5.

**Table 5:** Participants’ perspectives defining HRQoL (N = 22)

No.	Perspective	Frequency per participant	Contribution of the perspective to the overall definition of HRQoL
1.	Health status	11	HRQoL is the optimum level of health and freedom from dialysis with psychological and social wellbeing
2.	Socialization	9	HRQoL is the quality of social relationships, activities and support provided as well as the ability to play the expected social role within the family
3.	Psychological wellbeing	2	HRQoL is psychological wellbeing, including happiness and life satisfaction and the absence of psychological disturbance
4.	Religiosity	4	HRQoL is achieved through religious beliefs and behaviors in addition to physiological, psychological, and social wellbeing and financial security
5.	Financial income	4	HRQoL is the financial security of self and the family
6.	Needs satisfaction	3	HRQoL is the satisfaction of actual needs
7.	Healthcare services quality	2	HRQoL of patients undergoing hemodialysis is achieved through the quality of healthcare services provided in addition to physiological, psychological and social wellbeing

Some defined HRQoL in terms of health status and the freedom of restrictions imposed by the illness (renal failure) and its treatment (hemodialysis).

I have hypotension and fatigue and all the time I am exhausted, all the time I can’t work on that day (QI-06)

Other participants defined the concept in terms of socialization as having social relationships, participating in social activities, getting social support from others and playing the expected social role.

The most important is the family, and its stability. (QI-05)

Psychological wellbeing was used to refer to HRQoL, and terms such as accepting illness, being used for dialysis, satisfaction with life, happiness and hope were also used.

Of course, the psychological wellbeing, if the person is psychologically well, he will continue in life. And if the psychology is not well, he will become a little isolated. (QI-04)

Another aspect that was considered to define HRQoL was religiosity. Discussions included fatalism, belief in God, being close to God by performing religious worship and participating in charity work.

It increases my believe in Allah, it raises my morals; my psychology will be good, when I have that belief. I perform all my obligations as a Muslim, my prayers, all these things help me. (QI-12)

Financial income and the satisfaction of needs were also used in defining HRQoL independently, illustrating the subjectivity of the concept. Each participant discussed issues and concerns that were considered important. The achievement of those needs was considered to define the concept of HRQoL.

My financial status is difficult… but I’ve got to pay for transportation. My income doesn’t help me to rent a house because the fee is expensive. (QI-04)

As I told you, if I had my sight, I would do the things that I like. Now the only thing that I do is come for dialysis and go back home, whether I watch television or I chat with my family, that’s all. However, the things that I do, the kitchen, I can go and take water, but nothing else, my family makes it for me, there are things that I wish to have but I don’t tell my family about, I feel that I made them bored, because of what I ask, that’s why if my sight was there, it would be better for me, I could go and come. (QI-22)

The quality of healthcare services was recognized when defining HRQoL by patients undergoing hemodialysis and was added to other aspects of life to define HRQoL.

health services are important in our daily life. QI-21

The participants’ responses were synthesized, and an overall definition of HRQoL that includes the generated themes was developed:

HRQoL is personal satisfaction with health, social, psychological and financial status, religious performance and the provided healthcare services for patients on hemodialysis.

### Domains and indicators of HRQoL

Thematic analysis revealed the emerging themes that were considered by the study participants as domains that composed the concept of HRQoL. These are the physiological, social, psychological, religious and vocational domains. Each domain was determined by key indicators (Table 6). The assessment of those domains could reveal the effect of renal failure and hemodialysis on HRQoL.

**Table 6:** Domains and indicators of HRQoL

HRQoL domain	Frequency per participant	Indicator
Physiological domain	14	Physical ability
		Comorbidity
		Illness and treatment complications
		Pain and discomfort
		Sexuality
		Sleep
		Diet and weight control
		Medications
		Vascular access
Social domain	14	Social relationships
		Social activities
		Playing the expected social role
		Social support
Psychological domain	13	Feelings of low mood
		Anxiety
		Fear
		Body image disturbance
		Life satisfaction
Religious domain	8	Religious beliefs
		Religious worship
Vocational domain	1	Ability to work
		Effect of illness on working ability
		Employers’ support
		Financial security

#### Theme one: physiological domain

The physiological domain refers to health status in terms of the effect of the illness (renal failure) and its treatment (hemodialysis) on health. Symptoms and illness complications, sleep disturbance, pain, diet and fluid restrictions and vascular access were used to define that domain.

My illness increased, tiredness, dizziness, I can’t get out like before, I can’t go, I can’t attend gatherings, then from the many medicines that I take, I started to have shivering in my hands, I can’t tell you, many things. (QI-18)

#### Theme two: social domain

The social domain was defined as socialization determined by having social relationships, participating in social activities, playing the expected social role and getting effective social support from close family members.

Of course, without social relationships, a person can’t live. (QI-03)

Even some events and family gatherings I don’t go because I get tired. (QI-08)

The person thinks of securing his family, so if anything happens to him, his family would be secured. (QI-21)

She stood next to me supporting and caring … my wife is behind me, she keeps on following me up. (QI-09)

#### Theme three: psychological domain

The psychological domain refers to the psychological wellbeing of the patient undergoing hemodialysis and satisfaction with life and acceptance of illness. It is indicated by life satisfaction, feelings of low mood, anxiety, fear and body image disturbance. Fear included fear of the hospital and dialysis machine, fear of death, fear of the unknown, fear of disability and fear of losing job.

at these critical units where people whom we know and we like, dies in front of our eyes… so we start thinking, when is our turn? (QI-06)

all of my body is disfigured, here and there, look everywhere there is a disfigurement, and this what bothers me. (QI-20)

#### Theme four: religious domain

The religious domain was defined as the religious beliefs that help the patient on hemodialysis accept their illness and their ability to perform religious worship.

I believe in my destiny and God’s will. (QI-21)

Ok, throughout the interview I heard you mentioning many religious things, would these things affect the quality of life? [SILENCE] Of course, if the person is keeping his religion and following the religious doctrines, he will differ. But if he was mmm… I clean the Mosque, pray and attend the lessons in the Mosque, we like these things. QI-07

#### Theme five: vocational domain

Finally, the vocational domain was defined as the patient’s ability to work and the effect of illness on this ability. Financial security and employer support were also considered when defining the vocational domain.

I could not work in another place, as no company will accept that you work one day and have the other day off. (QI-05)

As much as the person improves his financial level, he will rest psychologically. (QI-21)

### Conceptualization of HRQoL

The concept of HRQoL was conceptualized based on patients’ needs and concerns and their own interpretation of HRQoL. In that analysis step, it was essential to identify the relationship between the domains of HRQoL and the overall concept of HRQoL. Before attempting to conceptualize HRQoL, a literature review on the conceptualization of QoL and critical appraisal of different models took place to identify the key domains of QoL [10–18]. The next step involved determining the life domains specified by the participants and identifying the relationships between them. Additionally, each domain had a weight that was estimated by reviewing and analyzing the participants responses which indicated that the psychological domain was centralized in the HRQoL model and was affected by all other domains, i.e., physiological, social, religious and vocational domains.

The religious and social domains directly contributed to HRQoL and are affected by the physiological domain, including the illness and its treatment. The physiological domain indicated by the chronicity of the illness and dependence on treatment affected the psychological and social domains and indirectly altered the religious and vocational domains.

Healthcare services were discussed during the interviews as an essential aspect of the patient’s life. The quality of those services affected the physiological wellbeing of the patient and were described as the gates to the way out of a miserable life that a patient on hemodialysis lives.

The coordination between healthcare services is important because this is one team, and the patient must enter from one gate and get everything. I expect that the good health and the good life come through these gates; if one of these gates were lost or got impaired, life wouldn’t be good. (QI-06)

Despite this need for healthcare services as an essential determinant of patient’s wellbeing, it was considered a factor contributing to HRQoL rather than a domain.

## Discussion

The concept of health-related quality of life (HRQoL) is a highly subjective nebulous term that is interpreted differently by people depending on their social, cultural and political backgrounds. Its actual meaning and conceptualization are lacking within the Saudi context, as there is little consensus on what it actually means. Hence, the current study aimed to explore the concept of HRQoL as interpreted by patients with end-stage renal disease undergoing hemodialysis in Saudi Arabia. This exploration provided an overall definition of the concept and identified its key domains that were structurally conceptualized.

### Definition and subjectivity of HRQoL

The subjectivity of HRQoL was illustrated in the study findings, as each participant defined the concept from his or her own perspective and what is considered important in their lives. This explains the different definitions that emerged during data analysis. This is congruent with Ferrans and Powers [19] conceptualization of QoL, who considered satisfaction with life domains of importance to the individual.

Different definitions for the concept of HRQoL emerged from the interviews. Participants defined the concept from different perspectives, including health status, socialization, psychological wellbeing, religiosity, financial income, needs satisfaction and the quality of healthcare services. This reflects the subjectivity of the concept.

In terms of health status, the majority of the participants defined HRQoL as the optimum level of health and the freedom from hemodialysis and its complications. Psychological wellbeing was another aspect that was used to define the HRQoL concept. Terms such as life satisfaction, achievement of life goals, happiness and hope were used repeatedly during the interviews. financial income, satisfaction of needs and the quality of healthcare services were also highlighted when defining the HRQoL concept. Need satisfaction with reference to Maslow’s hierarchy of human needs was used to conceptualize the QoL concept by Sirgy [20], who emphasized that needs satisfaction is the key element for a better QoL of societies. These findings are in line with several studies’ who defined QoL in terms of the effect of illness and its complications on different life domains [15, 19, 21–23],

Socialization was considered when defining the concept of HRQoL in terms of social relationships, social activities, social support and playing the expected social role. These terms reflect the Saudi cultural backgrounds where social relationships and bonding are important. Patients on hemodialysis are affected by their illness and its treatment, which prevents them from participating in social activities such as attending family gatherings and social celebrations. This approach of defining the concept of HRQoL with respect to socialization was not synthesized in the literature, as socialization was considered a domain rather than being utilized for defining the concept. However, social support was illustrated in the Ferrell, Wisdom [24] definition for QoL, which may be due to the target population of his study, i.e., patients with cancer, who need support from key persons in their lives to cope with their illness. Similarly, in the current study, patients on hemodialysis required social support from family members and friends to overcome difficulties they anticipate during their illness experience.

Religiosity was one of the emerging themes when defining the concept of HRQoL. This was observed when using religious concepts such as belief in God, destiny and fatalism. In addition, participants referred to the ability to participate in religious worship, such as praying, fasting and participating in charity activities. In contrast, the Western conceptualization of the concept of HRQoL included religiosity/spirituality as key domains but not merely the definition of the concept [19, 25]. This difference between this study finding and the literature might be due to the nature of Saudi culture, which is dictated by the Islamic religion. People practicing Islam believe that returning to religion is one of the methods to cure their disease and to accept and cope with their illness. Additionally, they start to prepare for death and the afterlife by becoming more religious. These Islamic beliefs might be the reason behind considering religiosity when defining HRQoL. This finding is congruent with the Western literature discussing the role of religion in coping successfully with health concerns [26] and the relationships between spirituality and QoL for patients on hemodialysis [27].

Several definitions emerged from the study findings that reflect the subjectivity of the concept. Considering the definitions in practice, it can be convenient to generate an overall definition that considers different aspects that were used to define the concept:

HRQoL is personal satisfaction with health, social, psychological and financial status, religious performance and the provided healthcare services for patients on hemodialysis.

That definition included all dimensions that were discussed by the study participants. Additionally, it reflects the subjectivity of the concept, as it views quality of life in terms of personal satisfaction with different life domains. This definition is in line with the Saudi Centre for Evidence-Based Healthcare [28], who considered quality of life as an outcome indicator of healthcare. Additionally, the center provided a definition for HRQoL, indicating that it is defined as physical, mental and social wellbeing [29].

### Conceptualization of HRQoL

The study findings revealed five domains to conceptualize HRQoL. These are the physiological, psychological, social, religious and vocational domains.

#### Theme one: physiological domain

The physiological domain theme was developed after discussing health status with the study participants. The health status was presented in terms of the effect of illness and its treatment on the general health of the patients. These include but not limited to physical ability, pain and sleep disturbance which are found by multiple publications in the literature [30, 31].

Many indicators for the physiological domain were found to be important and affect the quality of patients’ lives (Table 4). One of these indicators of the physiological domain is the physical ability, which reflects the patient’s ability to perform certain activities, such as walking, self-care, and climbing stairs. When interpreting those findings, the physiological domain with its indicators is similar to those in the Western QoL/HRQoL models [10, 17, 32, 33] and is considered as an essential domain assessed in several QoL instruments such as The Kidney Disease Quality of Life Short Form [34].

#### Theme two: social domain

Moving to the social domain of HRQoL, three determinants define this domain: social relationships and activities, playing the expected social role and social support. These indicators were discussed and considered in defining the concept of HRQoL. This might be due to the Saudi social background, which is characterized by the social system and strong family bonds, as reflected in the study by Al-Jumaih et al. [35]. In comparison with the Western literature, the social domain was identified in the City of Hope Model by Ferrell et al. [36] in terms of caregiver burden, role and relationships, affection/sexual function and appearance. The socioeconomic domain was considered by the Ferrans Model (1996), specifying social relationships and emotional support. The findings in the current study uncover a minor difference between the social dimensions defined in the Western QoL/HRQoL models and the social dimensions of the current study. One example of this difference is playing the expected social role, as gender variation appeared during the interviews. While men were concerned with securing their family’s financial needs, women held themselves responsible for family stability. These findings are in line with a study finding by Abdel-Khalek [37], who discussed the identified roles of men and women living in Arab countries. In contrast, the Western models lacked this gender variation when individualizing the concept of QoL/HRQoL.

Social activities, one of the indicators of the social domain, were discussed with reference to the Saudi cultural build-up that differs when compared with the Western community. Conversely, social support was discussed within the Western literature, as it is an expected social norm within different cultures. In Saudi Arabia, the social system obligates confinement to social responsibility among community members. This responsibility is in terms of social support physically and emotionally in addition to social relationships and activities.

#### Theme three: psychological domain

The psychological domain was identified by patients on hemodialysis and is determined by life satisfaction, feelings of low mood, anxiety, fear and body image disturbance. It was found that gender variation was apparent in the study findings, as female patients were concerned with body image disturbance, and male participants discussed their frustration caused by their failure to secure their family’s needs. Body image is a concern that affects patients on hemodialysis, which is supported in the literature by Muringai [38], Lin et al. [39] and Padilla [40].

Fear was also another psychological concern that was discussed by participating patients. Their concerns were fear from the unknown, fear of disability and dependence on others and fear of failing to secure family needs, which was obvious in male participants who held themselves responsible for their family members’ wellbeing. This finding is in line with findings of Lin et al.’s study. Furthermore, QoL/HRQoL Western conceptual models included fear as an indicator within the psychological domain [36, 41].

#### Theme four: religious domain

Religiosity was considered a key domain in the current study, and it was defined in terms of the ability to perform religious worship and religious beliefs that help patients cope with their illness and live a successful illness experience. Similarly, the Western QoL/HRQoL models considered religiosity/spirituality as a domain of the concept of QoL [13, 21, 36, 41]. Despite this similarity, the indicators differed from those in the current study. Examples of this variation is the use of terms such as “inner strength, conviction and life goals, faith in God and trust in God”. These differences may be due to the religious backgrounds of the Western communities where these models have been developed and conceptualized. Compared with the current study participants, whose religious background is Islam, spirituality is not a term in everyday language. Hence, it was not used when defining religiosity.

The second difference is the indicators of the religious domain that appeared in the current study. One indicator was religious beliefs, including belief in destiny, clearing sins through accepting illness and rewards in the afterlife. The other indicator was the ability to perform religious worship; for example, fasting is interrupted during the hemodialysis procedure. These beliefs and practices build up the religion of Islam. Hence, they were apparently expressed by the current study participants.

An interpretation of these findings is that the majority of the study participants practiced Islam as a religion and a way of life, and their responses to defining and conceptualizing the concept of HRQoL were influenced by their religious background, which helped them cope with their illness and live a positive life experience. However, there is a limited body of literature investigating religiosity and QoL and their relationships in Muslim communities; hence, interpreting this finding is difficult. Additionally, religious background must be considered when assessing QoL using instruments of Western origins in a Muslim community.

#### Theme five: vocational domain

The ability to work, employer support and financial security determined the vocational domain in the current study. The patient’s ability to work during hemodialysis is impaired due to their physical limitations. This impairment affected the sustainability of their jobs, as they either retired early or quit their jobs because they were unable to commit to a full-time career. These decisions are influenced by employers’ support, as patients working in the governmental sector in Saudi Arabia had better support than patients working in the private sector. Patients on hemodialysis are offered an off-day on their dialysis days without deduction of their salaries following the Royal order to consider their dialysis day as a paid off-day. However, this is practiced only in the governmental sector and not in the private sector. This affected their financial income and the financial security of self and family, as observed in male study participants in particular.

Socioeconomic indicators, including occupation, education and income, were considered in QoL/HRQoL models developed in Western communities [15, 17, 25, 41] with respect to the different socioeconomic statuses of Western countries and the governmental influence on that domain.

### Conceptualization of the HRQOL model for patients on hemodialysis in Saudi Arabia: renal-specific versus culture-specific domains

**Fig. 1:**
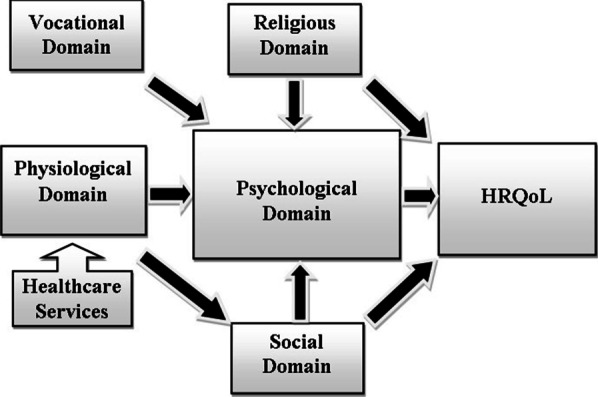
HRQoL conceptual model for patients undergoing hemodialysis in Saudi Arabia

The current study conceptualized the concept of HRQoL for patients on hemodialysis in Saudi Arabia (Fig. 1). Five domains defined HRQoL, and those domains were interrelated with each other and influenced HRQoL overall. In order to conceptualize the concept, the key domains were identified and the relationships between the domains were specified with the arrows direction. A review of existing QoL/HRQoL models was performed to guide our conceptualization.

It was found that the psychological domain was centralized within the model and was affected by the determinants of the other domains and it had a direct influence on HRQoL. There is a direct relationship between the religious, social, physiological and the vocational domains and the psychological domain. Furthermore, there is direct relationship between the psychological, social and religious domains and the HRQoL. This finding is supported by WHOQOL SRPB Group [42] study who found apparent relationships between SRPB and the psychological and social domains of QoL. Likewise, Abdel-Khalek [37] found a significant positive correlation of religiosity and subjective wellbeing and QoL. In contrast these relationships were lacking in some of the reviewed western models other than, Ferrell et al. [36], Grant et al. [16] and Wyatt and Friedman [13].

Furthermore, some of the domains were culture-specific, i.e., Saudi-specific. These included the social domain, the religious domain, and the vocational domain, which were apparently influenced by the Saudi cultural backgrounds, as discussed. Conversely, the physiological domain and the psychological domain are disease-specific (renal-specific) domains that are indicated by factors related to the disease and its treatment.

The difference between culture- and disease-specific domains is that the culture-specific domains are influenced by Saudi culture and will be similar in chronic illnesses other than renal failure, such as diabetes mellitus [43], hypertension [44] and hepatitis [45]. However, the disease-specific domains were generated from issues and concerns related to the disease process, i.e., renal failure. Examples are the physiological consequences of renal failure, such as sleep complications and illness manifestations, as fatigue is shared among patients with renal failure from different cultures. Similarly, the psychological domain is influenced by the disease and its treatment, as patients complain of depressive symptoms, but patients with bronchial asthma suffer from anxiety [46]. However, in the current study, anxiety was initiated from the fear of the unknown future. There is a need for culture-specific domains to define the concept of HRQoL within a specific society, as Kagawa-Singer et al. [47] determined the importance of culture in determining QoL, as culture describes the ways of achieving a good life and defines life within a set of values and beliefs. Furthermore, Corless et al. [48] emphasize that QoL instruments are valid within the social context where they have been developed. An example is the cross-cultural adaptation of the WHOQOL-100 instrument, which was developed after the exploration of the QoL concept across different cultures [49].

## Conclusion and recommendations

This study explored the concept of HRQoL as perceived by patients on hemodialysis in Saudi Arabia. The concept was defined, and the domains and key concepts were identified. It was apparent that Saudi culture influenced the concept definition and key domains illustrating the individuality and subjectivity of the concept and the effect of culture in understanding the concept. Hence, measuring the HRQoL of patients with ESRD on hemodialysis required a culturally adapted instrument that measures different aspects of patient life that are of importance to members of that culture. This would yield valid findings that could guide the planning of healthcare services to this group of patients. Additionally, the conceptualization of HRQoL in the current study yielded different meanings and understandings that are suitable to the Saudi context, which might be more convincing to healthcare providers and planners of care. Eventually, developing a culture-specific HRQoL instrument for patients on hemodialysis in Saudi Arabia or adapting an existing tool to allow for the comparison of findings among different cultures would be recommended.

## Supplementary Information

**Additional file 1:** COREQ (COnsolidated criteria for REporting Qualitative research) Checklist.

**Additional file 2:** Development of codes into categories and themes.

## Data Availability

The study data is available when required.

## Abbreviations

HRQoL: Health-related quality of life
PRO: Patient-reported outcome
ESRD: End-stage renal disease

## References

[1] Saudi Center for Organ Transplantation. Annual report for organ transplantation in Kingdom of Saudi Arabia. 2019.

[2] Dąbrowska-Bender M (2018). The impact on quality of life of dialysis patients with renal insufficiency. Patient Prefer Adher.

[3] Dejvorakul S (2019). Factors predicted with quality of life among hemodialysis patients in private hospital of Thailand. Hosp Pract.

[4] Coons SJ (1998). Reliability of an Arabic version of the RAND-36 Health Survey and its equivalence to the US–English version. Med Care.

[5] Al Garni RS (2015). Exploring the concept of health-related quality of life for patients with end-stage renal disease on haemodialysis in the Eastern Region of Saudi Arabia.

[6] Braun V, Clarke V (2006). Using thematic analysis in psychology. Qual Res Psychol.

[7] Meyrick J (2006). What is good qualitative research?. J Health Psychol.

[8] Krefting L (1991). Rigor in qualitative research: the assessment of trustworthiness. Am J Occup Ther.

[9] Caress A-L. Professor of Health Services Research, Department of Nursing and Midwifery, School of Human and Health Sciences, Director, Centre for Applied Research in Health.

[10] Carlozzi NE (2013). Impact of blood pressure dysregulation on health-related quality of life in persons with spinal cord injury: development of a conceptual model. Arch Phys Med Rehabil.

[11] Mathias SD (2008). Impact of chronic immune thrombocytopenic purpura (ITP) on health-related quality of life: a conceptual model starting with the patient perspective. Health Qual Life Outcomes.

[12] Ferrans CE (2005). Conceptual model of health-related quality of life. J Nurs Scholarsh.

[13] Wyatt GKH, Friedman LL (1996). Development and testing of a quality of life model for long-term female cancer survivors. Qual Life Res.

[14] Wilson IB, Cleary PD (1995). Linking clinical variables with health-related quality of life: a conceptual model of patient outcomes. J Am Med Assoc.

[15] Zhan L (1992). Quality of life: conceptual and measurement issues. J Adv Nurs.

[16] Grant M (1992). Measurement of quality of life in bone marrow transplantation survivors. Qual Life Res Int J Qual Life Asp Treat Care Rehabil.

[17] Ferrans CE (1996). Development of a conceptual model of quality of life. Sch Inq Nurs Pract.

[18] Moons P, Budts W, De Geest S (2006). Critique on the conceptualisation of quality of life: a review and evaluation of different conceptual approaches. Int J Nurs Stud.

[19] Ferrans CE, Powers MJ (1985). Quality of life index: development and psychometric properties. Adv Nurs Sci.

[20] Sirgy MJ (1986). A quality-of-life theory derived from maslow developmental perspective—quality is related to progressive satisfaction of a hierarchy of needs, lower order and higher. Am J Econ Sociol.

[21] Cella DF, Tulsky DS (1993). Quality of life in cancer: definition, purpose, and method of measurement. Cancer Investig.

[22] Cowan M, Graham KY, Cochrane B (1992). Comparison of a theory of quality of life between myocardial infarction and malignant melanoma: a pilot study. Prog Cardiovasc Nurs.

[23] Oleson M (1990). Subjectively perceived quality of life. J Nurs Scholarsh.

[24] Ferrell BR, Wisdom C, Wenzl C (1989). Quality of life as an outcome variable in the management of cancer pain. Cancer.

[25] Oleson M (1990). Subjectively perceived quality of life. Image J Nurs Scholarsh.

[26] Koenig HG, Larson DB, Larson SS (2001). Religion and coping with serious medical illness. Ann Pharmacother.

[27] Finkelstein FO (2007). Spirituality, quality of life and the dialysis patient.

[28] Saudi centre for evidence based health care. About us. 2014; Available from http://www.moh.gov.sa/endepts/Proofs/Pages/Definition.aspx.

[29] Schunemann H, et al. Saudi Arabian handbook for healthcare guideline development [Cited 2014]. 2014.

[30] Davison SN (2003). Pain in hemodialysis patients: prevalence, cause, severity, and management. Am J Kidney Dis.

[31] Gusbeth-Tatomir P (2007). Sleep disorders: a systematic review of an emerging major clinical issue in renal patients. Int Urol Nephrol.

[32] Mathias SD (2008). Impact of chronic immune thrombocytopenic purpura (ITP) on health-related quality of life: a conceptual model starting with the patient perspective. Health Qual Life Outcomes.

[33] Wilson IB, Cleary PD (1995). Linking clinical variables with health-related quality of life: a conceptual model of patient outcomes. JAMA.

[34] Hays RD (1997). Kidney Disease Quality of Life Short Form (KDQOL-SF), version 1.3: a manual for use and scoring.

[35] Al-Jumaih A (2011). A study of quality of life and its determinants among hemodialysis patients using the KDQOL-SF instrument in one center in Saudi Arabia. Arab J Nephrol Transpl.

[36] Ferrell B (1992). The meaning of quality of life for bone marrow transplant survivors part 1. The impact of bone marrow transplant on quality of life. Cancer Nurs.

[37] Abdel-Khalek AM (2010). Quality of life, subjective well-being, and religiosity in Muslim college students. Qual Life Res.

[38] Muringai T (2008). Dialysis access and the impact on body image: role of the nephrology nurse. Br J Nurs.

[39] Lin C-C, Lee B-O, Hicks FD (2005). The phenomenology of deciding about hemodialysis among Taiwanese. West J Nurs Res.

[40] Padilla GV, Grant MM (1985). Quality of life as a cancer nursing outcome variable. Adv Nurs Sci.

[41] Grant M (1992). Measurement of quality of life in bone marrow transplantation survivors. Qual Life Res.

[42] Group W.S (2006). A cross-cultural study of spirituality, religion, and personal beliefs as components of quality of life. Soc Sci Med.

[43] Al-Shehri AH (2008). Health-related quality of life in type 2 diabetic patients. Ann Saudi Med.

[44] Al-Ghamdi MS (2002). Quality of life in a sample of hypertensive patients attending primary health care facilities in Al-Khobar, Saudi Arabia. J Fam Community Med.

[45] Abdo AA (2012). Health-related quality of life of Saudi hepatitis B and C patients. Ann Saudi Med.

[46] Ten Thoren C, Petermann F (2000). Reviewing asthma and anxiety. Respir Med.

[47] Kagawa-Singer M, Padilla GV, Ashing-Giwa K. Health-related quality of life and culture. In: Seminars in oncology nursing. Elsevier; 2010. 10.1016/j.soncn.2009.11.008 20152579

[48] Corless IB, Nicholas PK, Nokes KM (2001). Issues in cross-cultural quality-of-life research. J Nurs Scholarsh.

[49] The Whoqol Group (1998). The World Health Organization quality of life assessment (WHOQOL): development and general psychometric properties. Soc Sci Med.

