# Supplementary material for: The concept of HRQoL for patients on hemodialysis in Saudi Arabia: an exploratory study
Source: Health Qual Life Outcomes. 2021 Dec 24;19:273. doi: 10.1186/s12955-021-01906-6 (PMC8709990; doi:10.1186/s12955-021-01906-6)
Supplement: Supplementary file 2 — Additional file 2: Development of codes into categories and themes. [file 12955_2021_1906_MOESM2_ESM.doc]

**Development of codes into** categories and themes

| No. | Code | Category | Theme |
| --- | --- | --- | --- |
|  | Ability to drive | Physical / cognitive ability | Physiological domain |
|  | Ability to fast | Fasting & Ramadan | Religious domain |
|  | Ability to travel | Travelling | Recreation |
|  | Ability to work | Vocational consequences of illness | Vocational domain |
|  | Acceptance | Coping | Psychological domain |
|  | Acceptance without satisfaction | Coping | Psychological domain |
|  | Accepting vascular access | Vascular access | Physiological domain |
|  | Activities after dialysis | Physical abilities | Physiological domain |
|  | Activities during dialysis | Dialysis session | Healthcare services |
|  | Activities on off-dialysis days | Physical abilities | Physiological domain |
|  | Adapting life to dialysis | Coping | Psychological domain |
|  | Adapting to illness & dialysis | Coping | Psychological domain |
|  | Adjustment of dialysis schedule during Ramadan | Fasting & Ramadan | Religious domain |
|  | Appreciates spouse support | Social support | Social domain |
|  | Attending late to dialysis during Ramadan | Fasting & Ramadan | Religious domain |
|  | Becoming committed to dialysis | Dialysis restriction | Illness restriction |
|  | Behaviour towards healthcare provider instructions | Healthcare provider | Healthcare services |
|  | Being passive at the beginning of the illness | Reaction to illness | Psychological domain |
|  | Being positive about dialysis | Reaction to illness | Psychological domain |
|  | Being responsible about health | Reaction to illness | Psychological domain |
|  | Body image disturbance | Body image | Psychological domain |
|  | Boredom during dialysis session | Dialysis session | Healthcare services |
|  | Can't avoid dates in diet | Reaction to diet restriction | Physiological domain |
|  | Can't tolerate medication side effects | Reaction to medications | Physiological domain |
|  | Can't trust healthcare provider | Healthcare provider | Healthcare services |
|  | Cares about children's education | Parental role | Social domain |
|  | Cause of hepatitis C infection | Illness complications | Physiological domain |
|  | Cause of medication noncompliance | Medication noncompliance | Physiological domain |
|  | Cause of sleep disturbance | Sleep disturbance | Physiological domain |
|  | Cause of social isolation | Social activities | Social domain |
|  | Causes of dietary noncompliance | Dietary noncompliance | Physiological domain |
|  | Causes of education interruption | Education interruption | Consequences of illness |
|  | Causes of fear | Psychological status | Psychological domain |
|  | Causes of happiness | Psychological status | Psychological domain |
|  | Causes of not fasting | Fasting & Ramadan | Religious domain |
|  | Causes of not traveling | Travelling | Recreation |
|  | Changes after illness | Changes after illness | Consequences of illness |
|  | Co-morbidity and QoL | QoL-determinant | Conceptualisation of QoL |
|  | Comparing life before and after dialysis | Changes after illness | Consequences of illness |
|  | Comparing status before and after illness | Changes after illness | Consequences of illness |
|  | Compliant with diet restriction | Dietary compliance | Physiological domain |
|  | Compliant with fluid restriction | Dietary compliance | Physiological domain |
|  | Conceptualisation of quality of life | QoL-concept | Conceptualisation of QoL |
|  | Concerned about children | Parental role | Social domain |
|  | Concerned about dialysis when travelling | Travelling | Recreation |
|  | Concerned about financial security | Financial concerns | Financial domain |
|  | Concerned about marriage | Social relationships | Social domain |
|  | Concerned about mother's feelings | Family role | Social domain |
|  | Concerned about spouse needs | Spouse role | Social domain |
|  | Concerned about vascular access complications | Vascular access | Physiological domain |
|  | Concerns about Hepatitis C infection | Illness complications | Physiological domain |
|  | Conflict between cultural norms and patient’s illness | Social support | Social domain |
|  | Consequences of diet restriction | Diet restriction | Physiological domain |
|  | Consequences of dietary noncompliance | Dietary noncompliance | Physiological domain |
|  | Consequences of disability | Disability | Physiological domain |
|  | Consequences of family separation | Family separation | Social domain |
|  | Consequences of hepatitis C infection | Illness complications | Physiological domain |
|  | Consequences of illness | Changes after illness | Consequences of illness |
|  | Consequences of lack of healthcare services for expatriates | Lack of services | Healthcare services |
|  | Coping method | Coping | Psychological domain |
|  | Coping with stress | Coping | Psychological domain |
|  | Current feelings | Psychological status | Psychological domain |
|  | Daily activities | Physical abilities | Physiological domain |
|  | Days becoming the same | Changes after illness | Consequences of illness |
|  | Determinants of quality of life | QoL-indicator | Conceptualisation of QoL |
|  | Dialysis arrangement during travelling | Travelling | Recreation |
|  | Dialysis as a duty | Personal view of illness | Psychological domain |
|  | Dialysis complications | Illness complications | Physiological domain |
|  | Dialysis day is lost | Changes after illness | Consequences of illness |
|  | Dialysis during travelling | Travelling | Recreation |
|  | Dialysis restriction | Dialysis restriction | Illness restriction |
|  | Dialysis services in Saudi Arabia | Dialysis service | Healthcare services |
|  | Diet and fluid restriction | Diet restriction | Physiological domain |
|  | Difference between vascular accesses | Vascular access | Physiological domain |
|  | Disability | Disability | Physiological domain |
|  | Disability and quality of life | QoL-indicator | Conceptualisation of QoL |
|  | Discomfort during dialysis | Pain & discomfort | Physiological domain |
|  | Discomfort during needle insertion | Pain & discomfort | Physiological domain |
|  | Distress due to financial status | Financial concerns | Financial domain |
|  | Distress due to sick daughter | Parental role | Social domain |
|  | Distressed due to current status | Psychological status | Psychological domain |
|  | Dry weight control | Weight & diet control, self-care | Physiological domain |
|  | Education interruption | Education interruption | Consequences of illness |
|  | Effect of dialysis duration on daily activities | Changes after illness | Consequences of illness |
|  | Effect of disability on social activities | Social activities | Social domain |
|  | Effect of dry weight control | Weight & diet control | Physiological domain |
|  | Effect of good relationships | Social relationships | Social domain |
|  | Effect of illness on family | Social consequences of illness | Social domain |
|  | Effect of illness on family relationships | Social consequences of illness | Social domain |
|  | Effect of illness on financial income | Financial consequences of illness | Financial domain |
|  | Effect of illness on health | Health status | Physiological domain |
|  | Effect of illness on life | Changes after illness | Consequences of illness |
|  | Effect of illness on religious behaviours | Religion & illness | Religious domain |
|  | Effect of illness on social relationships & social activities | Social consequences of illness | Social domain |
|  | Effect of illness on spouse | Social consequences of illness | Social domain |
|  | Effect of illness on travelling | Travelling | Recreation |
|  | Effect of illness on work | Vocational consequences of illness | Vocational domain |
|  | Effect of poor healthcare services | Poor healthcare services | Healthcare services |
|  | Effect of religious behaviours and beliefs | Religion & illness | Religious domain |
|  | Effect of weather condition | Heat & illness | Environmental weather |
|  | Emotional response to body image disturbance | Body image | Psychological domain |
|  | Emotional response to illness | Psychological consequences of illness | Psychological domain |
|  | Emotional response to vascular access | Vascular access | Physiological domain |
|  | Emotional response to vascular access complications | Vascular access | Physiological domain |
|  | Emotional status after dialysis session | Psychological status | Psychological domain |
|  | Employer support | Employer support | Vocational domain |
|  | Experience of dialysing at a different centre when travelling | Travelling | Recreation |
|  | Family & social gatherings | Social activities | Social domain |
|  | Family activities | Social activities | Social domain |
|  | Family encouragement | Social support | Social domain |
|  | Family support | Social support | Social domain |
|  | Family visits in another country | Social activities | Social domain |
|  | Family's emotional response to illness | Family support | Social domain |
|  | Family's reaction to illness | Family support | Social domain |
|  | Family's sympathy | Social support | Social domain |
|  | Fasting & Ramadan after illness | Fasting & Ramadan | Religious domain |
|  | Fasting during dialysis | Fasting & Ramadan | Religious domain |
|  | Fear from the future | Psychological status | Psychological domain |
|  | Fear from the hopital and the dialysis machine | Psychological status | Psychological domain |
|  | Fear of death | Psychological status | Psychological domain |
|  | Fear of disability | Psychological status | Psychological domain |
|  | Fear of losing job and financial income due to illness | Financial status | Financial domain |
|  | Fear of the unknown | Psychological status | Psychological domain |
|  | Feeling about dialysis duration | Psychological status | Psychological domain |
|  | Feeling about lack of social support | Social support | Social domain |
|  | Feeling about loss of control | Loss of control | Control |
|  | Feeling about time wasted or spent on dialysis | Psychological status | Psychological domain |
|  | Feeling about travelling restriction | Travelling | Recreation |
|  | Feeling anxious | Anxiety | Psychological domain |
|  | Feeling better after seeking psychological support | Psychological status | Psychological domain |
|  | Feeling depressed | Depression | Psychological domain |
|  | Feeling disabled | Disability | Physiological domain |
|  | Feeling distressed when other patient dies | Psychological status | Psychological domain |
|  | Feeling helpless toward family needs | Parental role | Social domain |
|  | Feeling helpless towards diet & fluid restrictions | Diet restriction | Physiological domain |
|  | Feeling humiliated due to illness | Psychological status | Psychological domain |
|  | Feeling responsible about family & children | Parental role | Social domain |
|  | Feeling unsecure if changes dialysis centre | Dialysis centre | Healthcare services |
|  | Feelings about dialysis | Psychological status | Psychological domain |
|  | Feelings about dialysis timing | Psychological status | Psychological domain |
|  | Feelings about diet restriction | Diet restriction | Physiological domain |
|  | Feelings about disability | Disability | Physiological domain |
|  | Feelings about family support | Social support | Social domain |
|  | Feelings about fluid restriction | Diet restriction | Physiological domain |
|  | Feelings about hospital admissions | Health status | Physiological domain |
|  | Feelings about hospital appointments | Health status | Physiological domain |
|  | Feelings about impaired memory | Psychological status | Psychological domain |
|  | Feelings about interrupted fasting | Fasting & Ramadan | Religious domain |
|  | Feelings about lack of social relationships | Social relationships | Social domain |
|  | Feelings about medication | Medication | Physiological domain |
|  | Feelings about other's sympathy | Social support | Social domain |
|  | Feelings about sexual impairment | Sexual impairment | Physiological domain |
|  | Feelings about sleep disturbance | Sleep disturbance | Physiological domain |
|  | Feelings about social gathering | Social activity | Social domain |
|  | Feelings about spouse support | Social support | Social domain |
|  | Feelings about the overall illness experience | Personal view of illness | Psychological domain |
|  | Feelings about vascular access | Vascular access | Physiological domain |
|  | Feelings about work status | Work satisfaction | Vocational domain |
|  | Feelings at the beginning of dialysis | Psychological status | Psychological domain |
|  | Feelings of guilt | Psychological status | Psychological domain |
|  | Feels being burden on others | Psychological status | Psychological domain |
|  | Financial status | Financial status | Financial domain |
|  | Fitting life to dialysis schedule | Coping | Psychological domain |
|  | Forced to quit work | Employer support | Vocational domain |
|  | Future plans | Hopes | Hopes |
|  | Getting used to diet restriction | Diet restriction | Physiological domain |
|  | Getting used to illness & dialysis | Coping | Psychological domain |
|  | Getting used to medications | Medications | Physiological domain |
|  | Getting used to needle prick | Pain & discomfort | Physiological domain |
|  | Getting used to time wasted on dialysis | Psychological status | Psychological domain |
|  | Getting used to transportation difficulty | Treatment requirements / transportation | Healthcare services |
|  | Getting used to vascular access | Vascular access | Physiological domain |
|  | Getting used to weather condition | Heat & illness | Environmental weather |
|  | Grief feelings | Psychological status | Psychological domain |
|  | Health awareness | Health awareness | Physiological domain |
|  | Health awareness and health status | Health awareness | Physiological domain |
|  | Health awareness and quality of life | QoL-determinants | Conceptualisation of QoL |
|  | Health behaviours | Health awareness | Physiological domain |
|  | Health concerns | Psychological status | Psychological domain |
|  | Health status after dialysis session | Health status | Physiological domain |
|  | Health status after starting dialysis | Health status | Physiological domain |
|  | Health status during Ramadan | Fasting & Ramadan | Religious domain |
|  | Healthcare provider support | Healthcare provider | Healthcare services |
|  | Healthcare services | Healthcare services | Healthcare services |
|  | Healthcare services for expatriates | Healthcare services | Healthcare services |
|  | Hepatitis C infection | Illness complications | Physiological domain |
|  | Hiding psychological stress from others | Psychological status | Psychological domain |
|  | Hospital visits & admissions | Health status | Physiological domain |
|  | Hot weather and dialysis | Heat & illness | Environmental weather |
|  | Hot weather and fluid restriction | Heat & illness | Environmental weather |
|  | Hypotension management | Illness complications | Physiological domain |
|  | Illness becoming ordinary & habitual | Coping | Psychological domain |
|  | Illness complications | Illness complications | Physiological domain |
|  | Illness restrictions | Illness restrictions | Illness restrictions |
|  | Impairment of quality of life | QoL-indicator | Conceptualisation of QoL |
|  | Importance of family support for kidney patient | Social support | Social domain |
|  | Importance of good healthcare services | Healthcare services | Healthcare services |
|  | Importance of money | Financial status | Financial domain |
|  | Importance of religious aspect | Religion & illness | Religious domain |
|  | Importance of social relationships | Social relationships | Social domain |
|  | Importance of social support | Social support | Social domain |
|  | Importance of transportation to dialysis patient | Treatment requirements / transportation | Healthcare services |
|  | Improvements after dialysis | Health status | Physiological domain |
|  | Improvements of life quality | QoL-indicator | Conceptualisation of QoL |
|  | Independence | Self-care | Physiological domain |
|  | Indicators of QoL | QoL-indicator | Conceptualisation of QoL |
|  | Individuality of quality of life | QoL-concept | Conceptualisation of QoL |
|  | Inner contradiction | Cognitive ability | Physiological domain |
|  | Intact memory | Cognitive ability | Physiological domain |
|  | Lack of sleep during dialysis | Sleep disturbance | Physiological domain |
|  | Leisure time activities | Recreation | Physiological domain |
|  | Life after dialysis | Changes after illness | Consequences of illness |
|  | Life becoming routine | Perception of life | Psychological domain |
|  | Life continues | Coping | Psychological domain |
|  | Life priorities | Perception of life | Psychological domain |
|  | Life satisfaction | Satisfaction | Psychological domain |
|  | Limitations imposed by illness | Illness restriction | Physiological domain |
|  | Limitations of physical activity | Physical abilities | Physiological domain |
|  | Living far from dialysis centre | Treatment requirements / transportation | Healthcare services |
|  | Losing interest in clothes and shopping | Psychological status | Psychological domain |
|  | Loss of control | Loss of control | Control |
|  | Making decisions about health | Control over health | Control |
|  | Meal changes during Ramadan | Fasting & Ramadan | Religious domain |
|  | Meaning of QoL | QoL-concept | Conceptualisation of QoL |
|  | Medication noncompliance | Medications | Physiological domain |
|  | Medications | Medications | Physiological domain |
|  | Medications side effects | Medications | Physiological domain |
|  | Memory impairment | Cognitive abilities | Physiological domain |
|  | Missing dialysis session | Dialysis commitment | Illness restriction |
|  | Missing family activities because of dialysis | Social activities | Social domain |
|  | Moving around | Physical abilities, illness restriction | Physiological domain |
|  | Noise & discomfort during dialysis | Dialysis session | Healthcare services |
|  | Noncompliance with diet restrictions | Diet restriction | Physiological domain |
|  | Optimism | Psychological status | Psychological domain |
|  | Pain & discomfort | Pain & discomfort | Physiological domain |
|  | Parental role | Parental role | Social domain |
|  | Personal view of illness | View of illness | Psychological domain |
|  | Personality before illness | Changes after illness | Consequences of illness |
|  | Personality change after illness | Changes after illness | Consequences of illness |
|  | Physical ability | Physical abilities | Physiological domain |
|  | Physical activity | physical abilities | Physiological domain |
|  | Problems of dialysis when travelling | Travelling | Recreation |
|  | Psychological disturbance due to illness | Psychological status | Psychological domain |
|  | Psychological status after illness | Psychological status | Psychological domain |
|  | Psychological status before dialysis | Psychological status | Psychological domain |
|  | Psychological wellbeing | Psychological status | Psychological domain |
|  | Psychological wellbeing & QoL | QoL-domain | Conceptualisation of QoL |
|  | Psychosomatic symptom | Psychological status | Psychological domain |
|  | Reaction to diet restriction | Diet restriction | Physiological domain |
|  | Reaction to family's way of support | Social support | Social domain |
|  | Reaction to hepatitis C infection | Illness complications | Physiological domain |
|  | Reaction to illness | Psychological status | Psychological domain |
|  | Reaction to vascular access | Vascular access | Physiological domain |
|  | Reasons for loss of control | Control | Control |
|  | Reassurance from others | Social support | Social domain |
|  | Relationship between financial status and psychological status | QoL-concept | Conceptualisation of QoL |
|  | Relationship between health status and psychological status | QoL-concept | Conceptualisation of QoL |
|  | Relationship between health status and social activities | QoL-concept | Conceptualisation of QoL |
|  | Relationship between healthcare provider care and patient's health behaviour | Healthcare provider | Healthcare services |
|  | Relationship between healthcare services and quality of life | QoL-concept | Conceptualisation of QoL |
|  | Relationship between the social domain and the psychological domain | QoL-concept | Conceptualisation of QoL |
|  | Relationship with children | Social relationships | Social domain |
|  | Relationship with dialysis centre | Dialysis centre | Healthcare services |
|  | Relationship with grandchildren | Social relationships | Social domain |
|  | Relationship with healthcare provider | Healthcare provider | Healthcare services |
|  | Relationship with spouse | Social relationships | Social domain |
|  | Relationship with the God | Religiosity | Religious domain |
|  | Relationships with other dialysis patients | Social relationships | Social domain |
|  | Religious aspect of illness | Religious domain | Religious domain |
|  | Religious domain | Religious domain | Religious domain |
|  | Satisfaction | Psychological status | Psychological domain |
|  | Satisfied with healthcare services | Healthcare services | Healthcare services |
|  | Scene inside the dialysis unit | Dialysis session | Healthcare services |
|  | Seek psychological support | Psychological status | Psychological domain |
|  | Seek to please the spouse | Social relationships | Social domain |
|  | Self-management | Control over health | Control |
|  | Sexual complications of illness | Sexuality | Physiological domain |
|  | Sleep disturbance | Sleep disturbance | Physiological domain |
|  | Social activities | Social activities | Social domain |
|  | Social domain of QoL | QoL-domain | Conceptualisation of QoL |
|  | Social isolation | Social activities | Social domain |
|  | Social relationships | Social relationships | Social domain |
|  | Social relationships before illness | Changes after illness | Consequences of illness |
|  | Social support | Social support | Social domain |
|  | Spouse emotional response to sexual impairment | Sexuality | Physiological domain |
|  | Spouse feelings about illness | Social support | Social domain |
|  | Spouse response to illness | Social support | Social domain |
|  | Spouse support | Social support | Social domain |
|  | Status during dialysis | Health status | Physiological domain |
|  | The need for family support | Social support | Social domain |
|  | The need for spouse support | Social support | Social domain |
|  | Thinking and decision making | Cognitive ability | Physiological domain |
|  | Thirst & fluid restriction | Diet restriction | Physiological domain |
|  | Thoughts of death | Psychological status | Psychological domain |
|  | Time wasted on dialysis | Dialysis session | Healthcare services |
|  | Time wasted on dialysis for expatriates | Dialysis session | Healthcare services |
|  | Transportation | Treatment requirements / transportation | Healthcare services |
|  | Transportation difficulty | Treatment requirements / transportation | Healthcare services |
|  | Travelling | Travelling | Recreation |
|  | Travelling restriction | travelling restriction | Recreation |
|  | Treatment at a far hospital | Treatment requirements / transportation | Healthcare services |
|  | Treatment difficulty for expatriates | Healthcare services | Healthcare services |
|  | Unsatisfied with dialysis in other dialysis centres | Healthcare services | Healthcare services |
|  | Vascular access | Vascular access | Physiological domain |
|  | Vascular access complications | Vascular access | Physiological domain |
|  | Weather condition | Heat & illness | Environmental weather |
|  | Weight control | Diet / control over health | Physiological domain |
|  | Willingness to fulfill family needs | Family role | Social domain |
|  | Wishes and hopes | Hopes | Hopes |
|  | Wishes to get a job | Hopes-vocational | Hopes |
|  | Wishes to get married | Hopes-social | Hopes |
|  | Wishes to have a salary | Hopes-financial | Hopes |
|  | Wishes to have children | Hopes-social | Hopes |
|  | Wishes to heal | Hopes-health | Hopes |
|  | Wishes to transplant | Hopes-health | Hopes |
|  | Wishes to travel | Hopes-recreation | Hopes |
|  | Wondering about the source of hepatitis C infection | Illness complications | Physiological domain |
|  | Work and dialysis schedule | Vocational consequences of illness | Vocational domain |
|  | Work satisfaction | Work satisfaction | Vocational domain |
